# Mandible Biomechanics and Continuously Erupting Teeth: A New Defect Model for Studying Load-Bearing Biomaterials

**DOI:** 10.3390/biomedicines9070730

**Published:** 2021-06-25

**Authors:** Jonathan Z. Baskin, Brandon M. White, Amit Vasanji, Thomas E. Love, Steven J. Eppell

**Affiliations:** 1 Department of Otolaryngology-Head & Neck Surgery, Cleveland VA Medical Center, Cleveland, OH 44106, USA; 2 Department of Otolaryngology-Head & Neck Surgery, Case Western Reserve University, Cleveland, OH 44106, USA; 3 Department of Biomedical Engineering, Case Western Reserve University, Cleveland, OH 44106, USA; bmw95@case.edu (B.M.W.); sje@case.edu (S.J.E.); 4 ERT Inc., Cleveland, OH 44114, USA; amit.vasanji@ert.com; 5 Population Health Research Institute, The MetroHealth System and Departments of Medicine and Population & Quantitative Health Sciences, Case Western Reserve University, Cleveland, OH 44106, USA; tel3@case.edu

**Keywords:** animal model, bone defect model, mandible biomechanics, elodont dentition, bone substitute material

## Abstract

Animals with elodont dentition and unfused mandible symphyses are hypothesized to have symmetric incisor morphology. Since these animals maintain their teeth by gnawing, they may provide physiologic feedback on mechanical function when unilateral mandible defects are created that manifest as ipsilateral changes in tooth structure. This defect model would potentially generate important information on the functional/mechanical properties of implants. Rats’ and rabbits’ mandibles and teeth are analyzed with µCT at baseline and post-intervention (*n* = 8 for each). Baseline incisors were compared. In a unilateral mandible pilot study, defects—ranging from critical size defect to complete ramus osteotomies—were created to assess effect on dentition (rats, *n* = 7; rabbits, *n* = 6). Within 90% confidence intervals, animals showed no baseline left/right differences in their incisors. There are apparent dental changes associated with unilateral defect type and location. Thus, at baseline, animals exhibit statistically significant incisor symmetry and there is an apparent relationship between mandible defect and incisor growth. The baseline symmetry proven here sets the stage to study the degree to which hemi-mandible destabilizing procedures result in measurable & reproducible disruption of dental asymmetry. In a validated model, an implant designed to function under load that prevents incisor asymmetry would provide supporting evidence that the implant has clinically useful load-bearing function.

## 1. Introduction

The larger project of which this study is a part is to develop a craniofacial load-bearing defect model that enables physiologic identification of the minimum mechanical properties required in a bone graft to reconstitute a defect in a highly stressed skeletal zone. This model would ideally possess the following attributes: (1) allow for an objective quantifiable measure of physiologically relevant mechanical function in bone, (2) provide longitudinal information on the function of a skeletal unit; function that is degraded by a defect but restored with skeletal reconstitution, (3) allows for the implantation of plates and/or load-bearing bone substitutes in the test (defect) region, and (4) be relevant and translatable to human applications. Animal models currently described in the literature such as those using implanted strain gauges, either fail to meet all of these criteria or introduce confounding variables that have been shown to alter the animal’s masticatory behavior [1]. We have identified an alternative method that uses a combination of mechanics and anatomical features unique to the teeth and jaws of some rodents and lagamorphs that may provide a more direct and reliable model.

**Figure 1:**
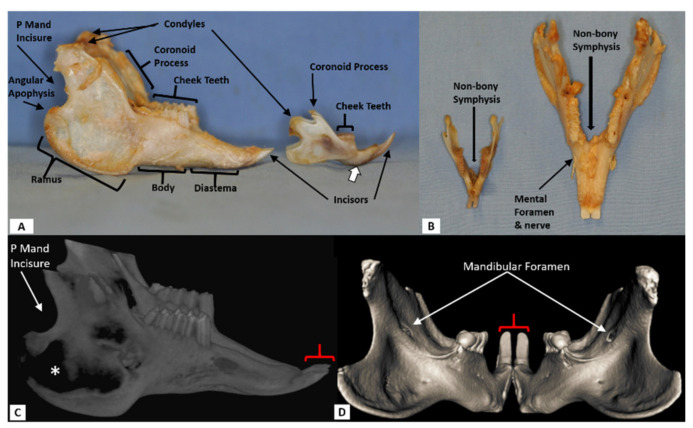
Non-defected adult male rabbit and rat mandible specimen. (**A**): Lateral view of the rabbit (left) and rat (right) compared side by side. (**B**): Dorsal (cranial) view (rabbit on right). Some of the features, which are characteristic of both animals, are only labeled on the rabbit mandible. The incisors are separated from the cheek teeth by a bony diastema that houses no teeth as canines are absent. The mandible body houses the cheek teeth (molars) and immediately posterior to the body is the ramus. Note that the reserve crown of the rat incisor, which can be discerned by the shape of the diastema (open white arrow in (**A**)), extends into the body under the cheek teeth. In contrast, the rabbit incisor reserve crown does not extend beyond the diastema. (**C**): 3D CT reconstruction of the rabbit mandible, lateral view. Note the incisor symmetry (red bracket). Volume averaging of the thin ramus bone results in the artifactual appearance of no bone (*). Also note that the condyles are partly cut out of the image. (**D**): CT reconstruction of the rat mandible, posterior (caudal) view showing the lingual surface of the hemi-mandibles and incisor symmetry (bracket). The mandibular foramen is noted which transmits the inferior alveolar neurovascular bundle. Note that the coronoid processes are cut out of the image. P Mand Incisure = posterior mandibular incisure (notch in between the condyle and angular apophysis).

The incisors of rats and the entire rabbit dentition are aradicular hypsodont (high crowned teeth with open roots) and elodont (continuously erupting). Putting aside the differences between rat and rabbit dentition (Figure 1), there are four common features making both rat and rabbit mandibles potentially good models to study load-bearing bone substitute materials. (1) These animals must constantly masticate and engage in gnawing activity to maintain proper tooth morphology in the continuously growing teeth. (2) Disuse of the elodont dentition is quickly reflected in uncontrolled growth and altered morphology of the erupted crowns (supra-gingival dentition) [2,3]. (3) The mandible is actually two hemi-mandibles that are joined by a non-bony fibro-cartilaginous symphysis (amphiarthrosis). While very stable, the fibrous symphysis articulates the two hemi-mandibles allowing the animal to rotate one side about the other. This angular movement, which can be up to 40° in the rat, enhances the animals’ ability to preferentially chew on one side of the mandible while leaving the contralateral side less mechanically loaded [4]. (4) Dental morphology can be accurately and non-invasively characterized via micro computed tomography (µCT). These properties are hypothesized to allow for an intervention that structurally weakens a hemi-mandible producing ipsilateral signs of neglect manifesting as altered morphology including but not limited to uncontrolled (longer) tooth growth ipsilateral to the defect. These changes can be quantified with µCT in real time.

Since it is expected that there will be non-negligible variability in tooth length between different animals (even of the same species and breed), it seems to make the most sense to develop a measure that compares the incisor ipsilateral to the defect to the contralateral incisor in the same animal. For example, the left and right incisors might be compared with the expectation that the incisor on the defected side would grow longer than that on the non-defected side. Before such an analysis can be made, it is necessary to determine if there is substantial left/right symmetry in the incisors of individual animals prior to creation of any defect. The existence of this symmetry is what the current paper intends to test using novel morphologic metrics.

In the rat and rabbit, a mandible critical size defect (CSD) is an established model for testing bone substitute materials [5,6,7,8]. A CSD in bone is one that will not heal on its own throughout the life of the animal without an intervention that is designed to replace or cause the growth of the bone volume. Prior work has demonstrated the dimensions of a CSD in the rat and rabbit mandible [5,6,7,8]. In the present study, all of the defects have dimensions that are at least as big as previously described CSD in their respective animals. Previously described CSDs for both animals have variable geometry and are small relative to the defect volume that is possible. To the knowledge of the authors, none have been specifically reported to affect dentition or mastication. Based on our intent to test load-bearing function, the mandible CSD appears to add little value beyond a CSD in a non-load-bearing site (such as the calvarium). To obtain in-vivo biomechanical information, some new defect model is needed that provides more information about mechanical and physiologic function. 

We have shown that bone grafts of precise dimensions can be press fit into critical size defects in the rat mandible with no implant mobility [5,9]. In the rabbit the geometry of the defect is similar, and it is expected that the same technique can be used. However, if not, there is sufficient bone in the rabbit mandible to enable the placement of hardware (plates and screws) that can secure implants. Also, it is envisioned that tissue or bone adhesives can be used (and possibly tested) to stabilize implants in the rabbit model.

To create a model that allowed for enough removal of bone to compromise the function of a hemi-mandible without destroying any nerve, vascular, or dental tissue, we chose to explore defects in the anterior ramus of the mandible. Since this was a pilot study (with respect to the defect model) and we were unsure of the defect size and geometry necessary to structurally weaken the hemi-mandible, we created a few different sized defects in each species. Below, we describe in some detail the surgical procedure used to create the defects. Since we only created defects of each size in one or two animals, we do not present any statistically significant results regarding changes in tooth morphology associated with these defects. We do, however, hypothesize that, under normal (no defect) conditions, aradicular hypsodont and elodont teeth in rats and rabbits are symmetric. We present a statistically significant response to this hypothesis.

## 2. Materials and Methods

### 2.1. Rat

The animal care and surgical procedure was performed in accordance with the guidelines of the Animal Resource Center (ARC) and IACUC at Case Western Reserve University (Protocol #: 08-16-JZB-RT1 04/22/2017). Eight male Sprague Dawley rats weighing ~700 gm were acquired (Charles River, Wilmington, MA, USA); for the sake of examining the presence of symmetry, 8 rats were studied and in the defect study, 7 animals were included. Prior to any intervention, animals were given a week after arrival at the ARC to acclimate to their new environment where they had access to a standard rat chow diet and water *ad libitum*. Study duration for defect healing of 3 weeks was chosen as a reflection of mandible bone healing in rats. 

#### 2.1.1. CT Scanning

Prior to imaging, the rats were anesthetized with an intraperitoneal injection of a standard anesthetic cocktail composed of ketamine hydrochloride (60 mg/kg) and xylazine (5 mg/kg). Rats were examined with μCT once prior to surgery, and then twice a week (every 3–4 days) for two and a half weeks during the study, and once following study conclusion at 3 weeks. µCT images (46 µm voxel resolution) of mandibles were collected as 720, 512 × 512 12-bit projection radiographs at 0.5° intervals around half the specimen.

#### 2.1.2. CT Image Processing

**Figure 2:**
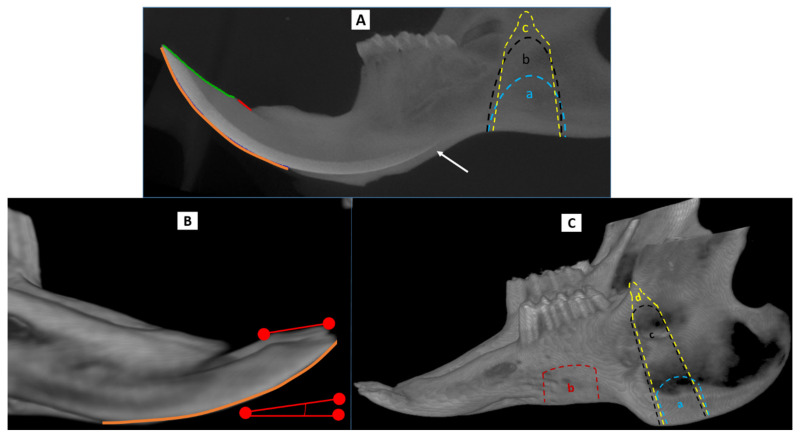
Incisor analyses of the rat and rabbit based on cone beam computed tomography (CBCT) and µCT. (**A**) Lateral view of a rat hemi-mandible: On the lingual (inner) surface of the incisor the short line (red) is a tracing of the path between the bony alveolus and occlusal (gnaw) ridge. The curved (green) tracing above marks out the path length of the occlusal surface (from gnaw ridge to tip) of the incisor. On the labial (outer) surface, the orange line traces the path length from the bony alveolus to the tip. Note the path of the incisor reserve crown (root) as it curves under the cheek teeth (arrow); more posteriorly it is not sufficiently mineralized to be radiopaque. In the ramus the defect designs are marked out; ‘a’ indicates the CSD, ‘b’ indicates the ID, and ‘c’ indicates the CRO. (**B**) Rabbit incisor: The path length of the labial surface was measured in the rabbit (orange tracing) as was the occlusal surface angle relative to the horizontal—mandibles from subsequent time points were registered to baseline scans as noted in the methods. (**C**) Defect design in the rabbit. The tracings indicate the CSD (a), body defect (b), ID (c), and CRO (d). Note that the CRO tracing for both the rat and rabbit more closely approximates the wide gap osteotomy but is not to scale. CBCT = cone beam computed tomography, µCT = micro computed tomography, CSD = critical size defect. ID = intermediate defect, CRO = complete ramus osteotomy.

Following acquisition and preprocessing, longitudinal reconstructed volumes were co-registered using an automated mutual information algorithm using simplex optimization developed in-house. Note that this type of registration uses all the pixels in an image rather than relying on specific landmarks. Volumes were visualized using MicroView (Parallax Innovations, Ilderton, ON, Canada) as well as VolNinja software (ImageIQ, Cleveland, OH, USA). µCT images of the animal skulls were cropped to include only the lower jaw. These cropped regions were skeletonized to provide the greatest bone and tooth detail. Incisor lengths were measured from bony alveolus (the bone that houses the teeth) to tip on the labial side, from bony alveolus to gnaw ridge on the lingual side, and the occlusal surface from gnaw ridge to tip (see Figure 2A). 

#### 2.1.3. Surgical Defect

Seven rats received defects. Prior to surgery, rats were anesthetized with the ketamine hydrochloride and xylazine cocktail. Unilateral mandible defects as described below were created on the left side allowing the contralateral unoperated side (right) to serve as a reference. A 15 mm linear incision was made along the inferior border of the left mandible. After the subcutaneous tissues were divided, the pterygomasseteric sling was incised at the inferior mandibular border. With minimal trauma to the muscles of mastication, the anterior ramus and posterior body of the mandible were exposed on both lingual and buccal sides in a subperiosteal plane preserving the periosteum. Defects in all animals were created using a medium speed drill with a 5 mm diamond burr (ANSPACH™ XMAX™ System) with continuous irrigation using normal saline. Surgery was performed using binocular magnification with care taken to identify and preserve the inferior alveolar neurovascular bundle (NVB) unless it was specifically targeted. Care was also taken to avoid entering the oral cavity. 

All mandibular defects in the rat were created in the anterior ramus just posterior to the tooth-bearing mandibular body (see Figure 2A). The location of defects at this site is limited anteriorly by the large reserve crown of the incisor, which extends under and posterior to the cheek teeth. Two rats received the smallest defect (using a 5 mm diamond burr), which was a critical size defect that measured 5 mm in diameter (Figure 2A blue outline labeled “a”). Three rats received the next larger defect (intermediate defect—ID) which was ~5 mm × 9 mm (Figure 2A black outline labeled “b”). This defect was intermediate in size between the CSD and a complete ramus osteotomy (CRO) and was designed to leave a mechanically robust strut of bone superiorly (dorsally) from which the intact coronoid process extends. The intermediate defect required vigilance to avoid damage to the reserve crown anteriorly, the mandible tail (angle) posteriorly, and the NVB superiorly. In two of the ID animals, the inferior alveolar NVB was preserved while in the other it was intentionally ligated. Two rats received complete ramus osteotomies (CRO) of the hemi-mandible (Figure 2A yellow outline labeled “c”)—one a narrow and the other a wide gap osteotomy. The CRO defect was identical to the ID except that the superior border of the ramus was osteotomized thereby unilaterally destabilizing the tooth-bearing mandible from the ramus. The narrow-gap CRO was a 1 mm gap designed with the intent of creating an environment where self-directed bony healing (with a bony union) was more likely. The wide-gap CRO entailed removing 3 mm of bone during the osteotomy in an effort to limit primary (direct) bone healing. The coronoid process was preserved in both CRO defects. Closure for all animals was meticulous and in two layers using resorbable suture. The muscles of mastication were resuspended, and the pterygomasseteric sling was reconstituted using 4-0 Vicryl (Ethicon, Summerville, NJ, USA), The skin and platysma were closed using 5-0 Monocryl suture (Ethicon, Summerville, NJ, USA).

#### 2.1.4. Postoperative procedures

Postoperatively, rats were given 3 doses of Kefzol and buprenorphine for analgesia. They were housed in individual cages and fed soft powdered diets which had the same nutritional composition as the standard diet for the first 3 days. Thereafter they were given a choice of powdered vs. standard rat chow. Each rat was euthanized 3 weeks after defect creation. The mandible was explanted with special care taken to leave the entire mandible intact. Explanted mandibles were scanned using µCT. 

### 2.2. Rabbit

Skeletally mature male New Zealand White Rabbits weighing between 2.8–3.2 kg were acquired from a USDA-licensed vendor (Covance Inc., Princeton, NJ, USA) and housed in the Case Western Reserve University Animal Resource Center (ARC). The research protocol was granted Institutional Animal Care and Use Committee approval (protocol # 2015-0056 (4/22/2017) at Case Western Reserve University. For the sake of analyzing baseline incisor symmetry, 8 animals were included in the study. Six animals were included in the defect study. A study duration of 10 weeks for defect healing was chosen to reflect the longer time frame for bone healing in the rabbit relative to rats [10]. Prior to beginning any work, the animals acclimated to their new environment in the ARC for at least one week. 

#### 2.2.1. CT Scanning

All animals were imaged prior to any intervention as a baseline study. For the animals in the defect aspect of the study, 3 additional CT scans were done: 1 week post-operatively, 3 weeks post-operatively, and 10 weeks post-operatively following euthanasia. The rabbits could not be imaged in the same µCT scanner as the rats because the rabbits were too large for the imaging chamber. Instead, they were imaged in a high-resolution cone beam computed tomography (CBCT) scanner (Carestream CS 9300). Prior to imaging, they were anesthetized by intravenous injection of midazolam and acepromazine and placed into a custom immobilizer to facilitate positioning within the CBCT scanner. Animals were scanned at both 180 μm and 90 μm voxel sizes with care taken to capture the entire mandible. The higher resolution imaging was used on the dentition to produce more accurate dental morphological analysis. Images were stitched together for the figures.

#### 2.2.2. CT Image Processing

Quantitative measurements of the right and left incisor were taken from the CT data. Mandibles were cropped and rotated so that the best-fit plane through the occlusal surfaces of the molars became the horizontal plane using Microview (version 2.5.0-rc21). The images were then transferred to *VolNinja* software (ImageIQ, Cleveland, OH, USA) which allowed CT images collected at later time points to be co-registered to baseline images using an automated mutual information algorithm developed in-house (as with the rats this type of registration uses all the pixels in an image rather than relying on specific landmarks). 3D incisor analysis and measurements were done with *Matlab version 2011B* (The MathWorks, MA, USA) and ImagePro version 7.0 (Media Cybernetics, Rockville, MD, USA), respectively. Measured aspects of the incisors were done in the sagittal plane (see Figure 2B). The path length along the labial surface of the incisor from the edge of the alveolar bone to the tip of the incisor was measured. The specific shape of the rabbit incisor made direct measurement of occlusal and lingual path lengths less reliable but allowed for another morphometric measure. The occlusal inclination angle of the erupted incisor crown, which also reflects tooth maintenance, was acquired and the angle between this and the horizontal (occlusal) plane was then compared between incisors at all time points. The molars were only measured in the rabbit with the mandible body defect. Measurements were from the edge of the bony alveolus to the tip of the highest buccal cusp. 

#### 2.2.3. Surgical Defect

Each of six rabbits were assigned to one of six distinct interventions. For five of the rabbits the defect location was similar to the rat; in the anterior ramus just posterior to the tooth-bearing mandible body (Figure 2C). The other defect was in the tooth-bearing mandible body and conforms to the conventional defect commonly used by investigators studying bone replacement materials in the rabbit mandible [11,12,13,14]. Rabbits were administered buprenorphine for analgesia and anesthetized by acepromazine and midazolam followed by intubation and administration of isoflurane during the procedure. The neck and submental region were shaved. An 18–20 mm unilateral linear skin incision was made in the skin overlying the inferior border of the left hemi-mandible. The pterygomasseteric sling was exposed and incised at the inferior mandibular border with minimal trauma to the muscles of mastication. The buccal and lingual surfaces of the mandible were exposed in a subperiosteal plane. As in the rats, the periosteum was preserved in the rabbits. Once the bone was exposed, a medium speed drill (ANSPACH™ XMAX™ System, Depuy Synthes, Warsaw, IN, USA) with a 5 mm cutting burr was used with continuous saline irrigation to create bony defects though smaller diamond burrs were used to complete the ramus osteotomy (see below). 

The six rabbit interventions were as follows: 2 rabbits with 8 mm x 8 mm CSDs, (Figure 2C, blue outline labeled “a”)—one rabbit with only a CSD the other had a CSD in conjunction with ligation of the ipsilateral NVB, one rabbit with a 10 mm x12 mm defect in the tooth-bearing mandible body located directly under the cheek teeth (Figure 2C, red outline labeled “b”), one rabbit with an intermediate defect (ID) (Figure 2C, black outline labeled “c”), measuring 23 mm in the ventral-dorsal dimension with a robust bony strut superiorly (dorsally) with the intact coronoid process extending from the lateral part of this strut, and 2 rabbits with CRO defects (Figure 2C, yellow outline labeled “d”); one with a narrow and the other a wide gap osteotomy. The CRO defect was the same as an ID but then extended to include an osteotomy through the superior (dorsal) strut just anterior to the coronoid process. The narrow gap consisted of a sharp 1 mm cut and was designed so as not to preclude innate self-directed bony healing whereas the wide gap osteotomy (3 mm) was intended to prevent direct bone healing (both preserved the coronoid process). In all cases (except when dictated by the experimental protocol), dental structures and the NVB were entirely preserved. Also, care was taken to avoid entering the oral cavity in all animals. Hemostasis was achieved using bipolar cautery. Photographs were taken of all defects prior to closure. Closure was meticulous and in 3 layers using resorbable suture. The muscles of mastication were resuspended, and the pterygomasseteric sling was reconstituted using 4-0 Vicryl (Ethicon, Princeton, NJ, USA), which was also used to close the platysma. The skin was closed using 5-0 Monocryl suture (Ethicon, Princeton, NJ, USA). 

#### 2.2.4. Postoperative Procedures

Rabbits were given postoperative daily antibiotics for 3 days and pain medications for 7 days, with subsequent doses given as needed. In addition to the normal high-fiber pellet feed, rabbits were offered (ad lib) softer foods such as kale and watermelon until pellet food was being consumed again at a normal rate. All animals were weighed weekly. Each rabbit was euthanized 10 weeks after defect creation. Mandibles were explanted with special care taken to leave the entire mandible intact. Explanted mandibles were scanned using CBCT.

### 2.3. Statistical analysis

Means of baseline measures (*n* = 8) of left and right incisors were compared without and with the assumption that individual measures were generated by a normally distributed process. 90% confidence intervals for the mean paired difference were generated by the bootstrap (normal distribution not assumed) and the paired t test (assumed a normal distribution). The effects of individual defects on tooth growth were described by Z scores specifying the degree to which individual measures deviated from expected means based on relevant baseline cohorts. To be clear, we are not claiming statistical significance of the tooth measures in defected animals. There were not enough replicates to support a test of significance. We simply present the Z scores as a means of describing how far from the baseline mean the tooth measures were relative to the standard deviation of the baseline 

## 3. Results

### 3.1. Rat Model 

All rats tolerated surgery well with no surgical complications and resumed a normal diet within 5 days with the exception of the full osteotomy defects (both narrow and wide gap osteotomies) which took around 10 days to resume a normal diet.

**Figure 3:**
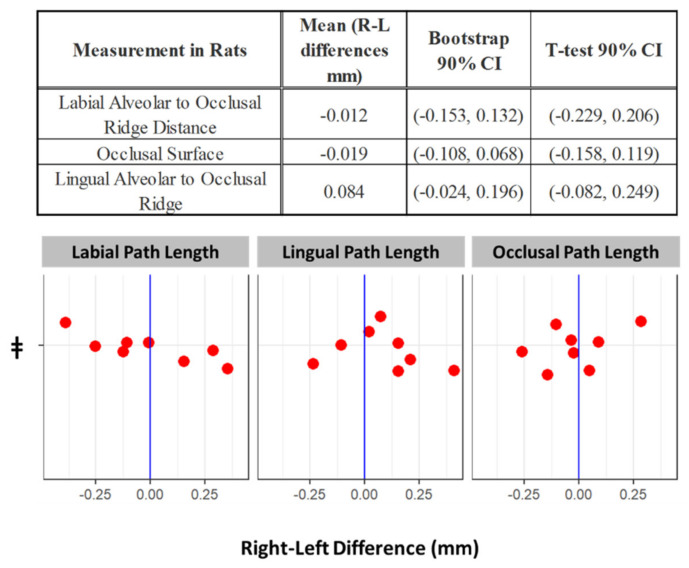
Difference between right and left rat incisors at baseline with 90% confidence intervals. The dots in the plot below describe the left-right differences of individual rats in the 3 different measures. ǂ Note that the vertical positions of the data points within each measurement category allow for better visualization of data but carry no meaning.

Based on the incisor metrics studied, the normal physiological condition of the incisors (the only teeth in rats that are elodont) is left-right symmetry (see Figure 3). Left vs. right labial (12.66 mm ± 0.59 vs. 12.63 mm ± 0.57) lingual (1.67 mm ± 0.25 vs. 1.60 mm ± 0.30) & occlusal (5.85 mm ± 0.51 vs. 5.87 mm ± 0.44) incisor path lengths displayed no differences within the 90% confidence interval as determined by both bootstrap and Student’s t-test analyses. Left and right sides similarly exhibited small variances.

**Figure 4:**
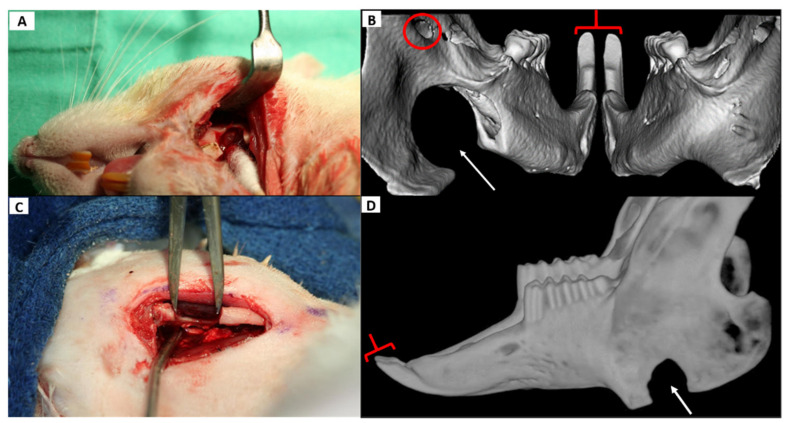
Critical size defect (CSD) in the rat and rabbit alongside reconstructed µCT & CBCT images. In both animals, the defect is in the anterior ramus. (**A**): Surgical creation of the CSD in the rat. The metal retractor is reflecting the masseter and skin and the Q-tip is medializing the medial pterygoid to show the 5 mm defect. (**B**): A 3D CT reconstruction of the rat mandible from a caudal (posterior) vantage point 3 weeks following the surgery. The defect (thin arrow) remained fairly unchanged and symmetric incisor morphology (brackets) can be seen which was present at all time points. The circle indicates the mandibular foramen which transmits the inferior alveolar NVB. (**C**): Surgical creation of an 8 mm CSD in the anterior ramus of a rabbit with caliper shown. (**D**): A lateral 3D CT reconstruction of the same rabbit mandible 10 weeks later (the caudal view used in the rat was not suitable in the rabbit as it did effectively reveal relevant anatomy in a single view.) Note the CSD (thin arrow) is largely preserved as is symmetric incisor morphology (bracket) which was maintained at all measured time points. CSD: critical size defect, µCT= micro computed tomography, CBCT= cone beam computed tomography, NVB = neurovascular bundle.

At no point postoperatively did the CSD (in either animal) exhibit noted bony regeneration in the defect volume determined radiographically (Figure 4B,C) or by visual evaluation of the explanted specimen. There were no changes in measured aspects of the ipsilateral incisor that extended beyond one standard deviation (in either direction) relative to baseline or the contra-lateral incisor at any time point. 

**Figure 5:**
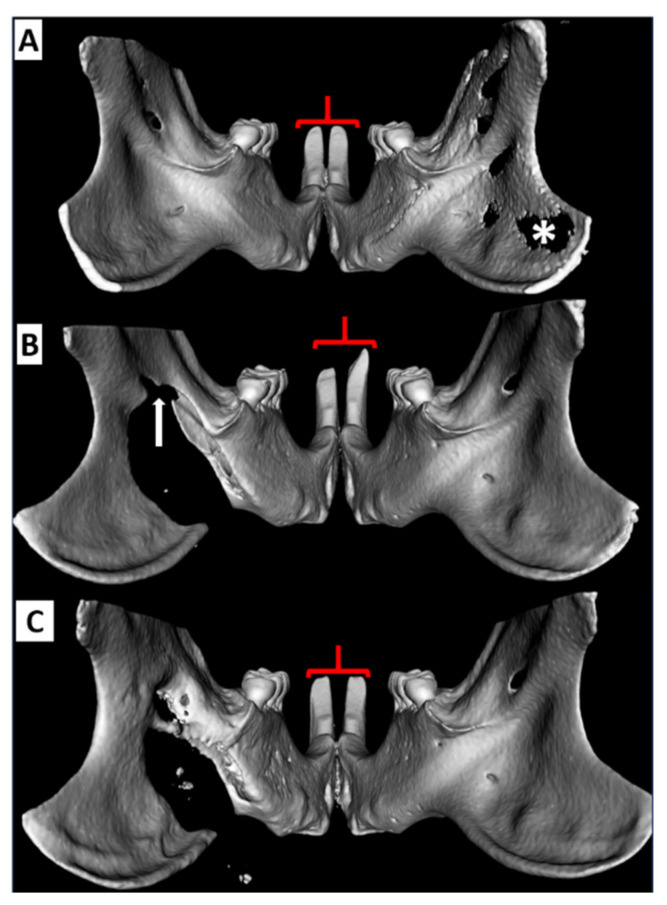
Reconstructed µCT images showing effects of ligation of the left inferior alveolar NVB & ID in the rat incisors: (**A**) Baseline image prior to defect creation. Note the incisor symmetry (red brackets). Volume averaging of the thin ramus bone on the right results in the artifactual appearance of no bone (*). (**B**) One week following defect creation and ligation of the NVB (arrow). Note in this case that there is bilateral incisor dysmorphology but the incisor contralateral (to the defect) is considerably longer. (**C**) Three weeks following defect creation there is complete restoration of incisor length and morphology. CBCT = cone beam computed tomography, µCT = micro computed tomography NVB: neurovascular bundle, ID: intermediate defect.

Similarly, the two animals receiving intermediate defects with intact NVB exhibited minimal defect changes and no observable changes in the ipsilateral incisor metrics relative to baseline or the contralateral side. The animal with an intermediate defect and concurrent ligation of the ipsilateral NVB exhibited effects on both incisors creating notable asymmetry (see Figure 5B). The contralateral incisor was longer while the ipsilateral incisor exhibited minor changes to the gnaw ridge. The most significant change was labial path length with the contralateral (right) incisor 1.7 mm longer than the left after one week (Z score of >2.5). This effect was entirely gone by 3 weeks with restored symmetry. 

**Figure 6:**
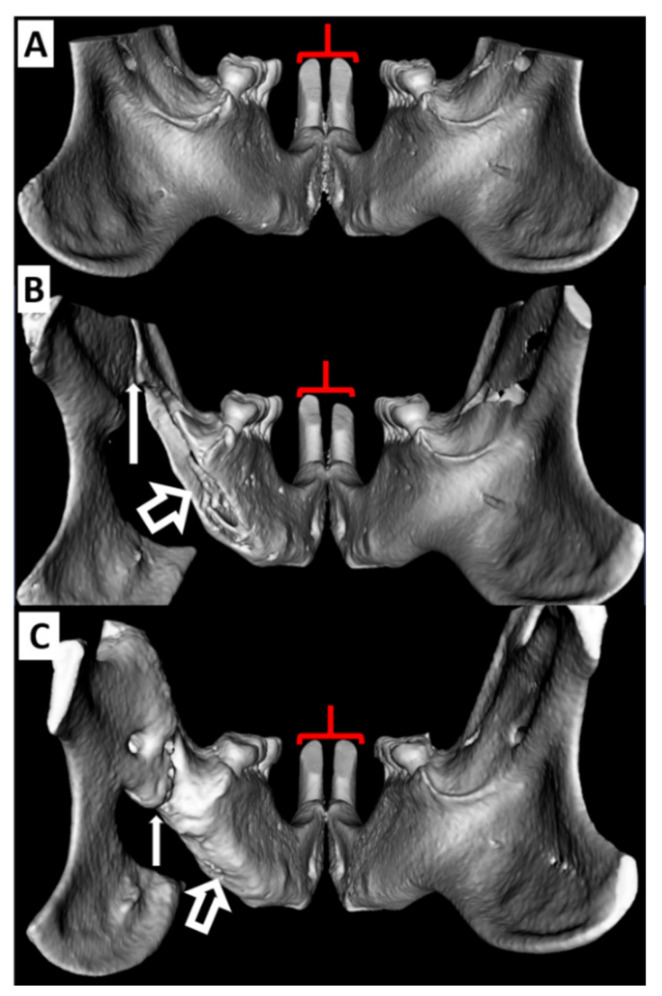
Reconstructed µCT images showing effects of left narrow gap CRO on rat incisors: (**A**) Baseline image. (**B**) One week following CRO (thin arrow). Note the incisor ipsilateral to the defect is longer (brackets). (**C**) At week 3 the defect has healed in a bony union (thin white arrow) in close association with restoration of incisor length and morphology. Note the bony deposition in the region of the reserve crown (compare open arrow in plate **C** to plate **B**). µCT= micro computed tomography, CRO = complete ramus osteotomy.

**Figure 7:**
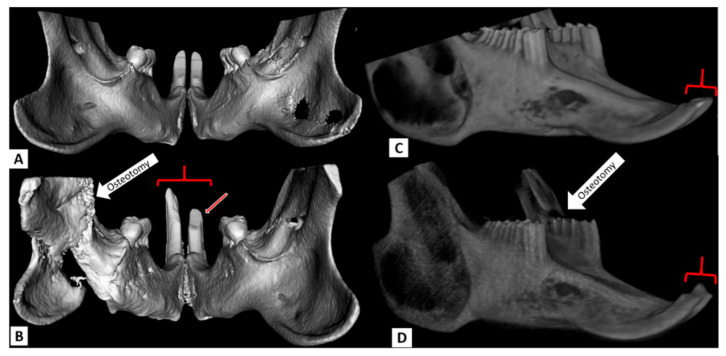
Reconstructed µCT & CBCT images of rat and rabbit mandibles with left side wide gap CROs: (**A**) Baseline rat image. (**B**) The rat mandible with the defect unhealed after the 3-week study conclusion: note the left incisor is considerably longer and dysmorphic with no recognizable occlusal surface morphology (brackets). The right incisor is slightly altered relative to baseline (arrow) but retains some of its occlusal surface morphology and length as the rat continued to masticate and gnaw on that side. (**C**) Baseline rabbit mandible image. (**D**) The rabbit mandible 3 weeks following CRO (arrow): Note the tooth ipsilateral to the defect is longer and the occlusal surface grossly altered relative to the right incisor (brackets). µCT = micro computed tomography, CBCT: cone beam computed tomography, CRO = complete ramus osteotomy.

The rat with a narrow gap CRO showed ipsilateral increase (relative to baseline) in incisor labial and occlusal path length measures of 0.85 mm and 0.40 mm respectively at one week (see Figure 6B). The left-right differences for this intervention are >2 standard deviations (Z score of >2) beyond the left-right differences seen in the baseline cohort. The defect was healed by 3 weeks in a bony union with concomitant restoration of normal morphology of the ipsilateral incisor and symmetry with the contralateral side. In the vicinity of the incisor reserve crown there was a large degree of bone deposition (see Figure 6C). The wide gap CRO resulted in uncontrolled ipsilateral incisor growth relative to the contralateral side. There was loss of a well-defined incisor gnaw ridge starting at 1 week and most prominently at the 3-week study conclusion (See Figure 7A,B) at which point the left incisor was 2.3 mm longer than the right using the labial measure (Z score >3). The mandible defect did not heal and the incisor growth (relative to both baseline and the contralateral side) continued to progress throughout the study duration. 

### 3.2. Rabbit Model

All rabbits tolerated the surgery well with no surgical complications. All rabbits whose defects did not result in a full osteotomy resumed a normal diet within 1 week. The 2 animals with full osteotomies took between 2 and 3 weeks to resume a normal diet. 

**Figure 8:**
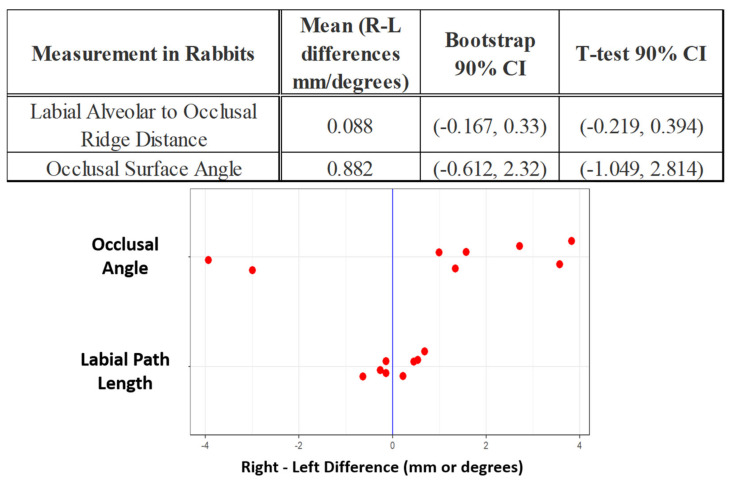
Difference between right and left rabbit incisors at baseline with 90% confidence intervals. The dots in the plot below describe the left-right differences of individual rabbits in the two different measures. ǂ Note that the vertical positions of the data points within each measurement category allows for better visualization of data but carry no meaning.

In the rabbit, the two metrics of tooth morphology also showed left-right incisor symmetry (see Figure 8). At baseline left and right labial path lengths (11.5 mm ± 1.1 vs. 11.6 mm ± 1.2) & occlusal angle (difference of 2.6 degrees ± 1.2) showed no significant differences.

**Figure 9:**
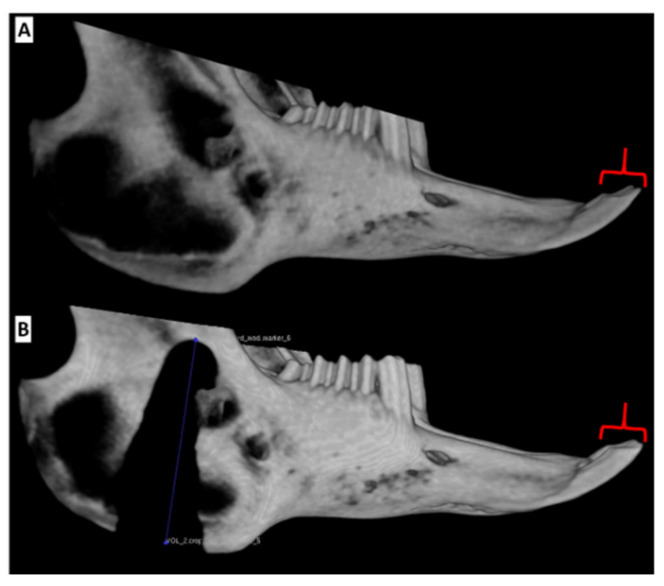
Reconstructed CBCT volumes of the rabbit right hemimandible ID. (**A**) Baseline image. (**B**) Image of the defected mandible 3 weeks following surgery. The line in the lower image measures the defect at 23 mm. The defect resulted in no changes of the ipsilateral incisor with retained morphologic symmetry (indicated by red brackets) between sides at all imaging time points. CBCT: cone beam computed tomography, ID: intermediate defect.

The CSD did not affect ipsilateral incisor morphology with retained incisor symmetry at all time points, and the defect volume showed little change after 10 weeks though the defect edges exhibited nominal remodeling (Figure 4C,D). The ID was readily created without harming dentition or the NVB. It also caused no notable changes in the ipsilateral incisor at any point with symmetry maintained between the sides (see Figure 9).

The rabbit receiving the defect and concurrent ligation of the inferior alveolar NVB, similar to the rat, exhibited bilateral incisor morphological changes that reversed during the study. The left and right occlusal angles changed by −9.5 and −7.6 degrees respectively which were considerably outside the 90% confidence interval of baseline angles (Z scores > 2 for both teeth). This corrected by 10 weeks on both sides. 

**Figure 10:**
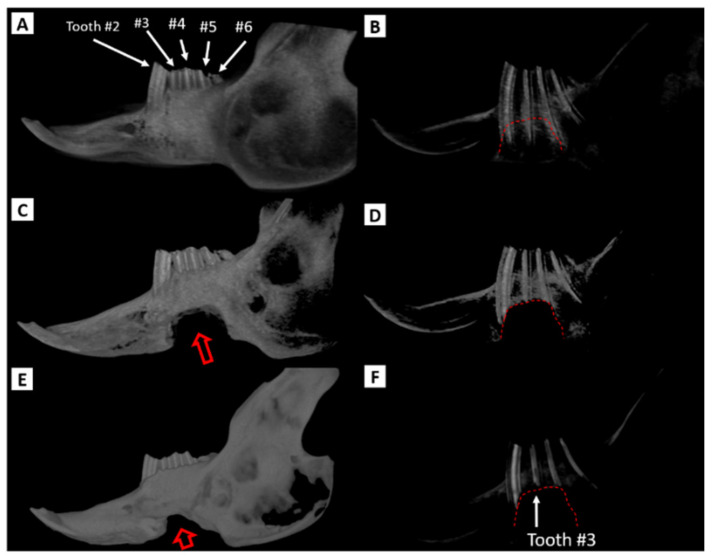
Reconstructed CBCT volumes of left rabbit mandible body defect (correlate with Table 1). The images on the left (**A**,**C**,**E**) highlight the surface anatomy of the left hemimandible. The images on the right (**B**,**D**,**F**) have been processed to display only the regions of high density thereby highlighting the cheek teeth (premolars & molars). (**A**,**B**): Left hemimandible prior to surgery. Teeth are labeled for orientation. Note the apices of the cheek teeth reserve crowns (roots) extend within millimeters of the inferior mandible border. (**C**,**D**): The hemimandible 1 week following the creation of a 10 × 12 mm defect along the inferior border of the tooth-bearing body of the mandible. (**E**,**F**): The hemimandible 10 weeks after defect creation. In (**C**,**E**) the red open arrow indicates the defect and in (**B**,**D**,**F**) the red dotted line outlines the original surgical defect. At 10 weeks the defect volume has decreased considerably (with bone deposition particularly notable in the vicinity of the reserve crowns) compared to one week while the erupted crowns appear shorter at 10 weeks than at baseline. Also note in image F the subtle resorption of reserve crown of tooth #3 in the 10-week scan (solid arrow). CBCT: cone beam computed tomography.

The mandible body defect under the cheek teeth, consistent in size and location with those reported in the literature, [13,14,15] is shown in Figure 10. The defect creation necessitated destruction of part of the cheek teeth reserve crowns (roots). At the last time point, there is apparent loss of viability in those teeth as reflected in the decreased length of the erupted crowns (see Table 1). At least one molar root (#3) also showed apparent atrophy beyond the original defect size at 10 weeks. The defect itself underwent considerable remodeling with loss of defect volume. In addition, in this body defect, there were bilateral incisor morphological changes with incisor occlusal angle changes of -5 and -9 degrees for left and right incisors respectively present at 10 weeks. These changes were considerable when compared to the baseline angles (Z > 2 for both).

**Figure 11:**
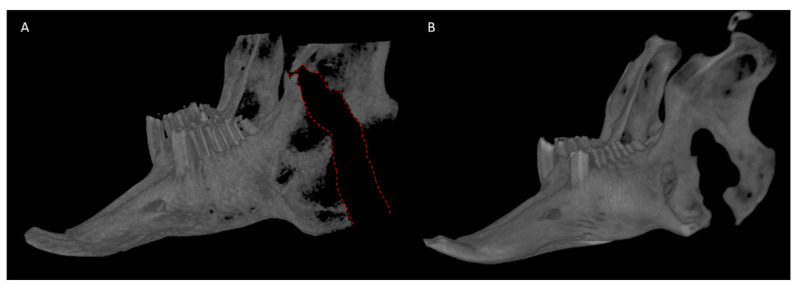
Reconstructed 3D CBCT volumes of rabbit narrow gap CRO. (**A**) Unhealed defect at 1 week; the red dashed line demonstrates the defect that extends through the superior ramus. (**B**) The same mandible after 10 weeks, the defect region has healed in a bony union with a concomitant return of normal incisor morphology and symmetry to the right side. CBCT: cone beam computed tomography, CRO: complete ramus osteotomy.

For defects that resulted in a total bony discontinuity between the ramus and body of the mandible, 1 week appears to be too soon in the rabbit (in contrast to rats) to see clear effects though the narrow-gap CRO is seen as a full fracture (Figure 11A). At 3 weeks the defect shows signs of bony healing with similar incisor lengths but asymmetric incisor occlusal planes. The left and right occlusal surface angle decreased by 17.9 and 9.8 degrees from the baseline, respectively (Z > 2.5). At 10 weeks, with the defect fully healed in a bony union, the incisors were symmetric and exhibited similar values to baseline in both measures.

The rabbit with the wide-gap CRO exhibited signs of ipsilateral neglect at 3 weeks (see Figure 7C,D). The incisor ipsilateral to the defect is >1 mm longer than the contralateral side (Z score of 2.5). The ipsilateral occlusal angle exhibited a >50 degree change compared to baseline (Z > 3). By 10 weeks there was complete restoration of incisor symmetry. However, the defect zone in the mandible did not appear fully ossified.

## 4. Discussion

With the goal of establishing a reliable animal model for evaluating load-bearing bone substitute and prosthetic materials, this study sought to explore the possible utility of an expanded mandible defect model in animals with aradicular hypsodont and elodont dentition—namely the rat and rabbit. The primary outcomes were based on µCT (or high resolution CBCT) data that generated reproducible and quantifiable measures of dental morphology based on the 16 animals (8 rats and 8 rabbits) studied. Based on the data presented, two questions can be answered with statistical significance. (1) Are the morphologic metrics of teeth as measured by µCT in this study reproducible measures by which to quantify tooth changes. (2) In the normal non-defected animal, does dental symmetry between the sides exist in aradicular hypsodont & elodont dentition? Three more questions present themselves, but further study is needed before they can be answered more definitively. (3) What is the ideal location of a mandible defect intended for testing biomaterials designed to provide load-bearing skeletal support while also protecting important structures (and thereby eliminating confounding variables)? (4) Can a properly sized unilateral mandible defect affect predominantly ipsilateral masticatory/gnawing function as manifested by dental morphological changes? (5) Is there a difference between the rat and rabbit that might compel selection of one over the other when designing preclinical animal trials? Finally, clinical translational potential is discussed as it relates to the defect model.

### 4.1. Are Morphological Tooth Measures as Assessed by High Resolution CT Reproducible?

Collecting µCT data on the rat was straight forward on a high-resolution small mammal CT scanner but it was more challenging for the larger rabbit. The full-size fan beam clinical scanners at our institution did not have the resolution necessary to allow for precise characterization of incisor morphology. We were able to generate relatively high-resolution images (90 micron voxels) in the rabbit using a CBCT scanner in the dental clinic. However, positioning challenges needed to be solved with a custom-made immobilizer given the designed clinical sitting configuration of the dental CBCT. Another issue is that most animal resource centers do not have access to CBCT scanners. A better option would be a medium sized animal scanner with high resolution (90 micron or better) that can accommodate rabbits and provide the images necessary for dental analysis. The specific dental metrics used for both the rat and rabbit are reproducible and effective at capturing small changes in incisor morphology. The small variances in the baseline metrics in both cohorts is an obvious indication of this. The CT image processing algorithms designed for this study produced output metrics based on dental anatomy that were repeatedly validated both by an experienced radiological analyst as well as an experienced clinician/surgeon in every single measure.

### 4.2. Does Left/Right Symmetry Exist in Normal Rat and Rabbit Tooth Morphology?

The issues of symmetric masticatory function and dental morphology are important in terms of understanding the results of this study. A validated assumption of symmetry would also have value in justifying use of the contralateral (non-defected) side as a control or reference point. Although symmetric function (and dentition) is intuitive, it should not be assumed to exist. In both the rat and rabbit, the mandible consists of two bones joined at the symphysis by fibrocartilaginous tissue which is common among mammalian clades (including some primates) [16]. There is consensus that the advantage of having an unfused symphysis is the degree to which it confers independent movement and function between the two hemi-mandibles during occlusion [17]. Therefore, given that these animals rely on masticatory function and gnawing activity to maintain their elodont dentition, asymmetrical dental morphology would seem entirely possible in the normal undisturbed state. However, within a 90% confidence interval, the baseline data presented here showed symmetric dentition in all measures of the laboratory rat and rabbit incisor. 

### 4.3. What Is the Ideal Location of a Mandible Defect?

If the objective is to maintain normal dental and masticatory function, the defect is best placed in the anterior ramus just posterior to the body of the mandible. In the rat, a body CSD would compromise the incisor reserve crown. In the rabbit, the defect we created in the mandible body not only permanently and unavoidably damaged cheek teeth in the immediate vicinity of the defect but also caused durable changes to both incisors that did not recover. This indicates masticatory function might have been permanently degraded on both sides. The anterior ramus location is ideal because it can avoid damaging dental structures and allows preservation of the NVB, maintaining direct neurovascular supply to the dentition and oral cavity. 

An interesting potential implication can be seen in prior studies [4,6,12] in which a mandibular body defect was created that likely involved some damage to the reserve crowns of the cheek teeth. These mandible body defects carry a high risk of disrupting the animal’s masticatory apparatus. This in turn could potentially compromise the mandible body defect model as a means for testing bone substitute materials if a claim of normal loading conditions is sought.

There is another potential problem with placing a defect in close proximity to reserve crowns (in either the rat or rabbit). In this study when the defect was contiguous with periodontal tissue, with healing there appeared to be greater deposition of new bone in the defect volume. This was seen in the rat with more rapid diminution of the defect volume that was exposed to the reserve crown of the incisor (Figure 6) and in the rabbit when the defect was exposed to the reserve crowns of the cheek teeth (Figure 10). Others have reported similar phenomena in rabbit mandible defects in close proximity to reserve crowns [11]. Due to the way elodont dentition is formed, such a defect is problematic if the intent is to test an implant with bone-forming potential. The regenerative and continual growth capacity of these teeth results, in part, from a pool of epithelial and mesenchymal stem cells that have the capacity to self-renew and differentiate into a variety of tissue-specific cell types [18,19]. Stem cells likely have regenerative capacity to become specialized dental tissue and also to differentiate into bone-forming cells. This presents a serious confounding variable when evaluating tissue engineered constructs for which hypotheses of osteoinductivity and osteogenic potential are often being tested. The same issue exists in the bone of the diastema which is in direct contact with the incisor reserve crowns in both rodents and rabbits. Researchers in the past have proposed critical size defect models in the rat mandible in this region [20]. This model too could be problematic if the intent is to test the inherent bone-forming potential of an implant. 

In addition to protecting dental structures, the two animals in which we ligated the NVB suggest that this structure should be protected as well. In the rat, NVB ligation resulted in ipsilateral dental morphological changes as well as distinct morphological changes to the contralateral tooth (see Figure 5). Similarly, in the rabbit, ligation of a unilateral NVB affected the bilateral incisors. Although the mechanism by which this effect was mediated cannot be determined from this study, based on the bilateral effect, sensory de-innervation may well play an important role. The effects of devascularization are expected to be limited to the ipsilateral side based on the vascular watershed area (inferior alveolar NVB). In both animals, it is noteworthy how quickly the dental changes reversed showing remarkable tissue resiliency and possibly a high degree of neural plasticity. Regardless of mechanism or reversibility, all defects in both the rat and rabbit presented can be created so as to spare the NVB thereby eliminating this confounding variable.

### 4.4. Relationship between Defect Volume/Type and Ipsilateral Dental Morphology

In both rat and rabbit, the CSD in the anterior ramus was not technically challenging to create. This defect caused no harm to important structures, did not fully remodel despite keeping the periosteum intact, and did not appear to impact dentition at any point following surgery. This last point indicates that the CSD does not produce a [physiologic] biomechanical effect. This corroborates past observations that the CSD could function as a negative surgical control with a consistent, reproducible result [5,8]. 

The larger [intermediate] defect in the rat mandibular ramus presents some challenge in terms of avoiding injury to important structures. The space in which to place a defect is relatively small—the trajectory of the reserve crown in the mandible has it curving posteriorly (caudally) and superiorly (dorsally) abutting the anterior ramus limiting the anterior (rostral) extent of the defect. Though there is some room to work superiorly while protecting the NVB, there is little room for error working posteriorly as any extension of the defect in this direction risks fracture of the tail (angle) of the mandible. Despite the challenge, it can be created in the rat and, like the CSD, caused no changes to the dentition. In the rabbit the intermediate defect is easily extended to over 20 mm (extending superiorly from the inferior mandible border) without injuring the dentition or the NVB. This defect also appeared to have no impact on the incisor growth at any point examined by CT. In both animals, eating and masticatory recovery with these larger-than-CSD defects (ID) occurred in the same time frame as animals with the smaller CSD. 

The CRO caused unmistakable changes in both the rat and rabbit with distinct loss of normal morphology of the ipsilateral incisor including longer ipsilateral tooth growth (relative to baseline and the contralateral incisor). In both animals, the preserved morphology in the contralateral incisor indicates that the animal is able to continue using that side. The continued use was consistent behaviorally with continued mastication and food consumption (albeit with a softer diet for the rabbits in the first 2 weeks). Also, both rat and rabbit with narrow gap CRO demonstrated complete bony healing of the defect with apparent synchronous restoration of incisor morphology (and symmetry). As expected, the healing and dental changes occurred more quickly in the rat than the rabbit. In the rat, the wide-gap CRO did not heal and resulted in irreversible (in the study time frame) and dramatic elongation of the ipsilateral incisor with relative maintenance of the contralateral morphology. Somewhat surprisingly in the rabbit wide-gap CRO, was the restored incisor symmetry (at 10 weeks) despite the fact that the defect repair zone did not appear fully ossified. The symmetry indicates an apparent return of functional use. It is not clear whether a fibrous non-union in the defect zone was undergoing a delayed healing process or had already healed in a bony union that was interrupted by a re-fracture. A larger study with a longer duration, more frequent imaging, and histopathological analysis of the mandible specimen is needed to better examine this. The explanted mandible specimens in this study were subjected to 4-point mechanical testing (not described in this manuscript) which rendered them poorly suited for histological fixation.

Therefore, based on the critical size and intermediate defects in the anterior ramus, there was no correlation between defect volume and tooth morphology. As the defect volume increases (particularly for rats), the risk of neural vascular bundle damage increases. If such damage occurs, this does result in changes in tooth morphology. If the defect is a complete osteotomy between the ramus and mandible body, then ipsilateral tooth morphology is clearly impacted. Based on this, we conclude that, in the anterior ramus, defect volume alone does not seem to affect tooth morphology assuming bony continuity remains between the ramus & body and both dental tissue and NVB are protected. To induce tooth morphological changes (aside from directly injuring dental tissue) it is necessary to either damage the neurovascular bundle or cause a frank fracture of the mandible that separates the tooth-bearing mandible from the ramus to which the muscles of mastication largely insert.

This study justifies further exploration of a full osteotomy defect model in both rats and rabbits for developing load-bearing bone substitutes, bone adhesives, or prosthetics such as resorbable plating systems. The unique dentition of these animals allows for real time feedback on the functional biomechanics of an implanted hemi-mandible. The pre-defect dentition serves as a baseline reference, and related to the non-bony symphysis, there is some evidence in this study that the contralateral (non-defected) side can also serve as a reference for functional mastication. If validated these models could help identify the minimum mechanical requirements that a synthetic bone substitute, adhesive or plate must possess to provide load-bearing skeletal support in the mandible. 

An important limitation of this study is related to study size. This is particularly true for the defect model development. Further studies will be necessary examining each individual defect and its associated effect on dentition in sufficient sample size to generate reliable and reproducible data. Another limitation is the lack of histomorphometry as a means to better understand the underlying biology. For example, to better understand these models for testing bone substitute materials, future studies would ideally be informed by an examination of bone healing and vascular watersheds. Finally, mechanical testing of the specimen would also yield important information and this work is ongoing. 

### 4.5. Is the Rat or the Rabbit a Better Model?

Advantages of the rat are lower cost and convenience which includes ease of housing and care. Related to its high metabolic rate, the incisors grow more quickly than they do in rabbits—its incisors can grow as much as 1 mm/day as opposed to 2–3 mm/week in rabbit incisors [2]. The fast healing time in the rat can be an advantage with reduced study length and costs but any preclinical study using the rat mandible model must take into account the relatively accelerated time frame [21]. 

There are indications that the rabbit mandible model, despite the greater expense and other inconveniences, would be more suitable. Most obviously, the rabbit mandible is physically larger than the rat making surgical procedures less technically demanding. Anatomically, the rabbit incisor reserve crown does not extend beyond the diastema which is anterior to the reserve crowns of the cheek teeth. The defect in the anterior ramus can easily be created without injuring the reserve crowns of any teeth. In contrast, the rat incisor reserve crown extends fully under the cheek teeth and abuts the anterior ramus making it more challenging, though certainly not impossible, to create the defect without causing some dental damage.

While the focus of this study was on rabbit incisors, rabbit cheek teeth are also continuously growing, allowing for additional sources of data compared to the rat which has brachyodont cheek teeth that do not continuously erupt. We focused only on incisors due to their rate of growth. Typically, rabbit incisors grow about 2–3 mm/week whereas the cheek teeth grow about 3 mm/month [22]. Therefore, a study of the cheek teeth would yield more data on functional jaw use, but it would require a longer study duration. 

Importantly, rabbits appear to exhibit basic multicellular unit remodeling that is naturally occurring (like humans), while rats have limited intra-cortical bone remodeling in the absence of external stimuli [23] Rabbits, as compared to rats, have cortical bone modulus and strength more similar to humans [24]. Also, it is possible in rabbits to harvest iliac bone for autogenous grafting which is valuable as a positive control when studying bone substitute materials.

Another reason to consider the rabbit as a strong preclinical model for testing load-bearing bone substitute materials stems from studies of the temporomandibular joint which have indicated similarities in the masticatory apparatus between rabbits and humans [25,26]. The orientation of the masseter in rabbits resembles the orientation seen in humans. Rabbit chewing patterns are intermediate between the rat and other ungulates (such as pigs). They have diverse jaw movement with rostral-caudal, dorsal-ventral, as well as lateral movements. Its chewing pattern involves loading and balancing the sides similar to humans [25,26]. Campillo et al. note, as a difference, the content of rabbit bone marrow to be higher in fat than human bone marrow and, cites as a concern, the affect this could have on bone healing [27]. However, there does not seem to be conclusive evidence that this translates into practical healing issues in the craniofacial skeleton. Aerssens et al., in their analysis of interspecies differences in bone, proposes that canine bone might offer the best likeness to human bone for studying bone substitute materials [28]. However, the cost of working with canines and the issues increasingly being raised about working with companion animals are making canines less practical as preclinical models, particularly in the interest of addressing a theoretical advantage. Working with rabbits is far less expensive and can be practical even in fairly large numbers. Mini Pigs are potentially good models particularly given the canines (tusks) that grow throughout life, but they are more expensive and challenging to manage [29].

### 4.6. Clinical Translational Issues

A validated animal model described here could also enable a more precise preclinical examination of requisite geometric properties for dental implantology. For instance, prior work on patients receiving dental implants that were 4 by 4 mm (as opposed to 8.5 mm long implants) exhibited four month results clinically comparable to the longer implant [30]. However, long-term results are not known, and preclinical models would be helpful in answering that question. On a more basic science level, this model can be used in conjunction with other described models to understand underlying dental and skeletal pathophysiology. For instance, this study touches on the underlying biology of the regenerative capacity for dental growth. There are other studies that seek to understand the mechanobiology between cancer cell and extracellular matrix interactions [31,32]. This model can potentially be used to shed further light on these questions.

## 5. Conclusions

µCT and high resolution CBCT are effective means for non-invasive quantifiable assessment of dental morphology in both rat and rabbit. The continuously erupting teeth in these laboratory animals exhibit left-right symmetry in the normal state. The rabbit, due to size and anatomy, is more amenable as a model. Creating a defect larger than a 5 mm CSD in the rat while preserving vital dental and neurovascular structures is possible but requires more skill. There appears to be a relationship between hemi-mandible stability and ipsilateral tooth growth in elodont dentition. In order to validate this as an animal model for in-vivo mechanical strength testing, future work on appropriate sample sizes and utilizing the dental metrics developed here will need to add more frequent imaging as well as histopathological analysis of mandible specimens. 

## Figures and Tables

**Table 1:** Length of cheek teeth in rabbit body defect. Measurements are only of the first 4 cheek teeth. The 5th tooth (#6) is not measured. Defect does not extend beyond tooth #4.

Analysis of Cheek Teeth Height (mm) in Rabbit with Mandible Body Defect								
Time	#2		#3		#4		#5	
	L	R	L	R	L	R	L	R
**Baseline** **(predefect)**	5.3	4.8	3.4	3.6	4.1	3.9	3.0	3.6
**One week**	5.4	5.2	3.7	3.6	3.5	3.8	3.8	3.9
**10 weeks**	3.8	5.3	2.1	3.8	3.0	3.8	3.2	4.0

Tooth #6, the last cheek tooth (molar), is not included in this analysis. Corresponds to rabbit shown in Figure 10.

## Data Availability

All relevant data is included in this article. Raw image data is available upon request to the corresponding author.
